# Investigating Changes in Pharmacokinetics of Steroidal Alkaloids from a Hydroethanolic *Fritillariae thunbergii* Bulbus Extract in 2,4-Dinitrobenzene Sulfonic Acid-Induced Colitis Rats

**DOI:** 10.3390/ph17081001

**Published:** 2024-07-29

**Authors:** Ji-Soo Jeong, Jeong-Won Kim, Jin-Hwa Kim, Eun-Hye Chung, Je-Won Ko, Youn-Hwan Hwang, Tae-Won Kim

**Affiliations:** 1 BK21 FOUR Program, College of Veterinary Medicine, Chungnam National University, 99 Daehak-ro, Daejeon 34131, Republic of Korea; jisooj9543@gmail.com (J.-S.J.); lilflflb@gmail.com (J.-W.K.); jinhwa926@g.cnu.ac.kr (J.-H.K.); ksissb1293@gmail.com (E.-H.C.); rheoda@cnu.ac.kr (J.-W.K.); 2 Herbal Medicine Research Division, Korea Institution of Oriental Medicine, Deajeon 34054, Republic of Korea

**Keywords:** pharmacokinetics, colitis, *Fritillariae thunbergii* Bulbus, alkaloids

## Abstract

*Fritillariae thunbergii* Bulbus (FTB), a member of the Liliaceae family, has a long history of use in many herbal formulations for traditional and modern clinical applications to treat various infections and inflammation. To understand FTB’s diverse physiochemical properties, it is important to determine the pharmacokinetic properties of its active constituents, the steroidal alkaloids. The aim of the present study was to investigate the pharmacokinetic alterations of the alkaloids, the active components of FTB, in the presence of colitis. A single oral dose of FTB (1 g/kg) was treated to a 2,4-dinitrobenzene sulfonic acid (DNBS)-induced colitis rat model to assess whether the colitis condition could influence the pharmacokinetics of the major alkaloids present in FTB. Among the four major alkaloids, peimisine exhibited a significantly increased systemic exposure, approximately five times higher, under the colitis condition compared with the normal state. Meanwhile, peimine, peiminine, and sipeimine exhibited shorter half-lives in the DNBS group without significant changes in systemic absorption. As herbal medicine may contain active substances with different or opposing efficacies, careful consideration of pharmacokinetic changes in individual components due to diseases is necessary. Further experiments on peimisine are required to ensure the effectiveness and safety of FTB’s clinical application in the presence of colitis.

## 1. Introduction

Steroidal alkaloids are nitrogenous derivatives that are secondary metabolites derived from natural plants, including Solanaceae, Liliaceae, and Fritillariae, and are recognized as potential biological effectors with anticancer, anticholinergic, antimicrobial, and anti-inflammatory activities [1]. *Fritillariae thunbergii* Bulbus (FTB), the bulb of *Fritillariae thunbergii* Miquel (Liliaceae), has a long history as a traditional herbal medicine used as an antitussive and expectorant in several Asian countries, including Korea, China, and Japan, as it is non-addictive and has fewer side effects than commercial morphine-based cough medications containing codeine [2,3]. FTB is used in many herbal formulae for traditional and modern clinical applications to treat a variety of infections, for mucus removal, pain relief, and inflammation [4,5].

A surge in scientific efforts has uncovered the multifaceted physiological activities of FTB by focusing on the identification of its active components [6,7]. The type and content of these steroidal alkaloids vary slightly depending on the extraction solvent and method used for FTB. The phytochemical characterization of ethanol-extracted FTB has been confirmed by previous studies which identified steroidal alkaloids including peimine, peimisine, and peiminine—the most abundant alkaloids detected—as active ingredients of FTB [6,7,8,9]. Among the biological properties of FTB-derived steroidal alkaloids, anti-inflammatory and analgesic effects have garnered significant attention. FTB has been reported as used traditionally for oral and gastric ulcers because of its anti-inflammatory effects [10]. Lee et al. [11] reported the effectiveness of FTB hydroethanolic extract, standardized with steroidal alkaloid components, in mitigating intestinal damage in a dextran sulfate sodium (DSS)-induced colitis mouse model, and confirmed that FTB can be used clinically for preventive purposes.

Medicinal and aromatic herbs contain diverse classes of active substances with different physiochemical properties, and there is a growing interest in exploring the active ingredients in the crude natural sources and in producing scientific evidence to fully understand medicinal plants for their further use [12]. In parallel with this growing interest, determining the pharmacokinetic features of active ingredients is largely accepted to improve the therapeutic potential of natural products [2,13]. The absorption of orally administered drugs can be influenced by the condition of the intestine, which can vary among individuals. Pathophysiological changes in patients with intestinal diseases can also affect outcomes and drug product performance. In particular, in ulcerative colitis, which is characterized by persistent and consistent inflammation that affects both the colon and rectum and involves periods of relapse and remission, inflammation-associated changes in intestinal permeability can exacerbate this variability, directly influencing the efficacy of drugs and potentially exacerbating side effects [14].

In oriental herbal medicine, which is often administered for preventive purposes or over extended periods, it is crucial to consider potential fluctuations in substance absorption arising from a disease state. Against this background, we assessed the alterations in the pharmacokinetics of steroidal alkaloids following oral administration of FTB in the presence of colitis.

## 2. Results

### 2.1. Steroidal Alkaloids Contents in FTB

**Figure 1:**
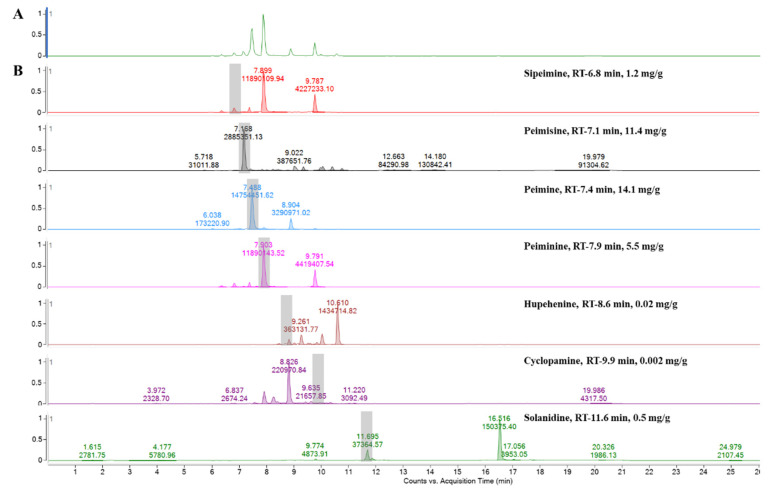
Ultra performance liquid chromatography–electrospray ionization–mass spectrometry analysis of (**A**) hydroethanolic *Fritillariae thunbergii* Bulbus extract and (**B**) reference standards.

**Figure 2:**
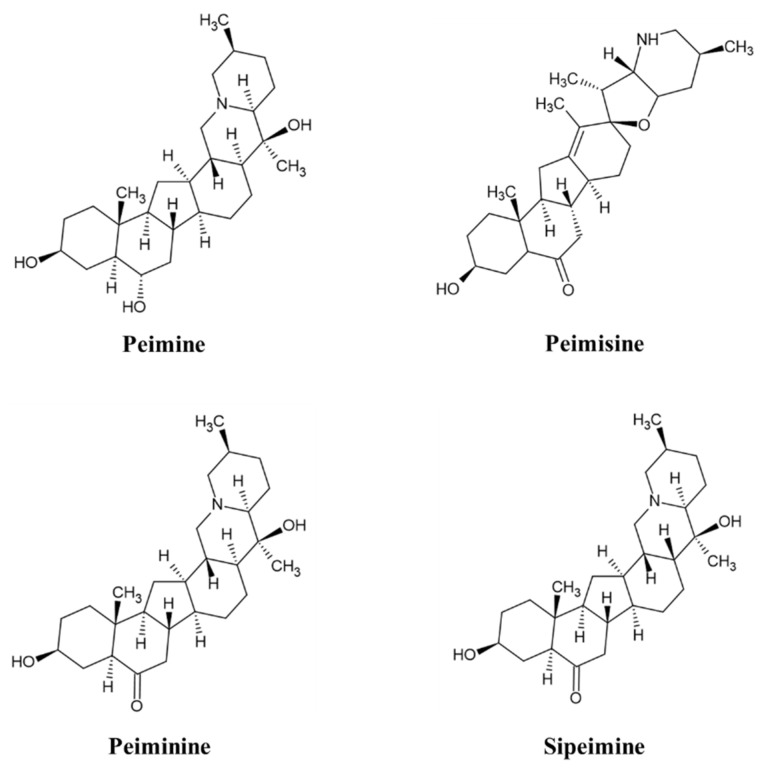
Chemical structure of peimine, peimisine, peiminine, and sipeimine.

Analyzed by comparing the retention time and peak matching of each reference standard, seven phytochemicals were detected in FTB. The monitored compounds were quantitatively analyzed using the external standard calibration method. Among the contents analyzed, peimine (14.1 mg/g), peimisine (11.4 mg/g), peiminine (5.5 mg/g), and sipeimine (1.2 mg/g) were found to be over 1 mg/g (Figure 1). The chemical structures of these four substances are shown in Figure 2.

### 2.2. The Anti-Inflammatory Effect of Active Ingredients in FTB on Lipopolysaccharides (LPS) Stimulated RAW 264.7 Cells

**Figure 3:**
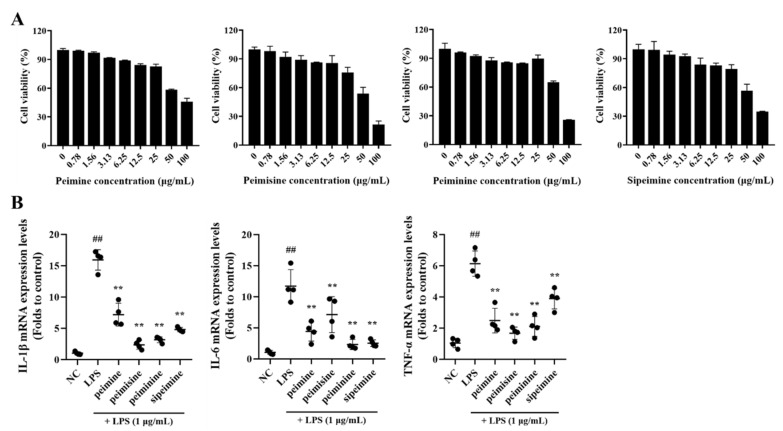
Cell viability and the anti-inflammatory effect of peimine, peimisine, peiminine, and sipeimine in RAW 264.7 cells. (**A**) Cell viability. (**B**) Interleukin (IL)-1β, IL-6, and Tumor necrosis factor (TNF)-α mRNA expression levels. NC, non-treated cells; LPS, stimulation of lipopolysaccharides (1 μg/mL); peimine, peimisine, peiminine, and sipeimine, LPS + peimine, peimisine, peiminine, and sipeimine 25 μg/mL, respectively. Values: mean ± SD (*n* = 4). Significance: ## *p* < 0.01 vs. NC; ** *p* < 0.01 vs. LPS by Dunnett’s multiple comparisons test, respectively.

None of the four major alkaloids in FTB, peimine, peimisine, peiminie, and sipeimine, showed any toxicity at concentrations of 0–25 μg/mL in RAW 264.7 cells (Figure 3A).

After treatment with each of the four compounds at non-toxic concentrations (25 μg/mL), the mRNA expression levels of pro-inflammatory cytokines in LPS-stimulated RAW 264.7 cells were reduced. This did not occur when cells were treated with LPS alone. The extent of the reduction varied by substance: Interleukin (IL)-1β and tumor necrosis factor (TNF)-α expression were lowered about 6-fold and 3-fold, respectively, when treated with peimisine, and IL-6 was lowered approximately 5-fold when treated with peiminine (Figure 3B).

### 2.3. Comparison of Symptoms and Colon Histological Damage to Normal in 2,4-Dinitrobenzene Sulfonic Acid (DNBS)-Induced Colitis in Rats

**Figure 4:**
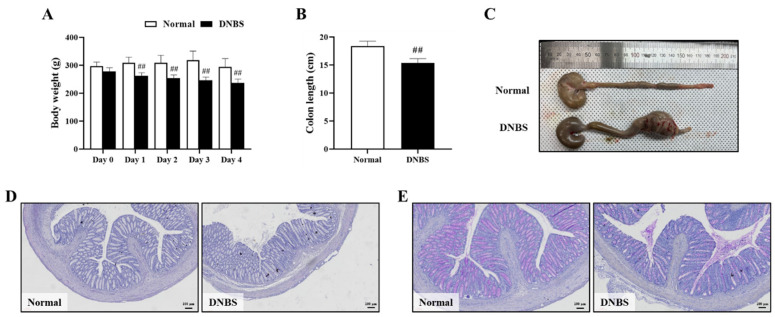
Comparison of phenotypes and colon histological injury to normal colons in 2,4-dinitrobenzene sulfonic acid (DNBS)-induced colitis in rats. (**A**) Body weight (g), (**B**) Colon length (cm), (**C**) Representative photographs of the entire colon, (**D**) Hematoxylin–eosin staining (Bar = 100 μm), and (**E**) Periodic acid-Schiff staining (Bar = 100 μm). Normal, normal control rats; DNBS, DNBS-treated rats. Values: mean ± SD (*n* = 5). Significance: ## *p* < 0.01 vs. Normal by unpaired *t*-test.

The DNBS-treated group exhibited diarrhea with significant weight loss on the day after administration, compared with the normal group (Figure 4A). In addition, the length of the colon was also significantly shortened when compared with the normal group (Figure 4B,C).

In colon histopathologic analysis, hematoxylin–eosin (H&E) staining showed inflammatory cell infiltration and severe intestinal epithelial crypt damage. Periodic acid–Schiff (PAS) staining confirmed goblet cell depletion in the colons of the DNBS-treated group (Figure 4D,E).

### 2.4. Differences in Pharmacokinetic Parameters of FTB Steroidal Alkaloids between Normal and DNBS-Treated Rats

**Figure 5:**
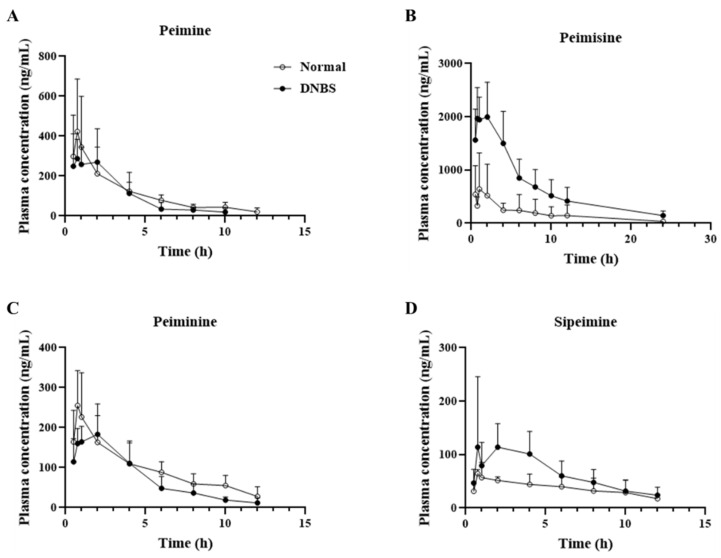
Mean plasma concentration vs. time profile of (**A**) peimine, (**B**) peimisine, (**C**) peiminine, and (**D**) sipeimine following oral administration of *Fritillariae thunbergii* Bulbus at a single dose of 1 g/kg to normal (-○-) and 2,4-dinitrobenzene sulfonic acid (DNBS)-treated (-●-) rats. Data expressed as mean ± SD (*n* = 5) for each time point.

As shown in Figure 5 and Table 1, a comparison of the pharmacokinetic parameters of the normal and DNBS-treated groups for the four major ingredients revealed that, unlike the other alkaloids, peimisine showed a significant increase in maximum plasma concentration (C_max_; Normal, 655.5 ± 671.9 ng/mL; DNBS, 2330.5 ± 363.2 ng/mL), and area under the curve (AUC; Normal, 3959.3 ± 4738.6 h∗ng/mL; DNBS, 6895.4 ± 5782.1 h∗ng/mL), indicating the extent of the drug’s exposure in the body under DNBS administration when compared to normal conditions.

**Figure 6:**
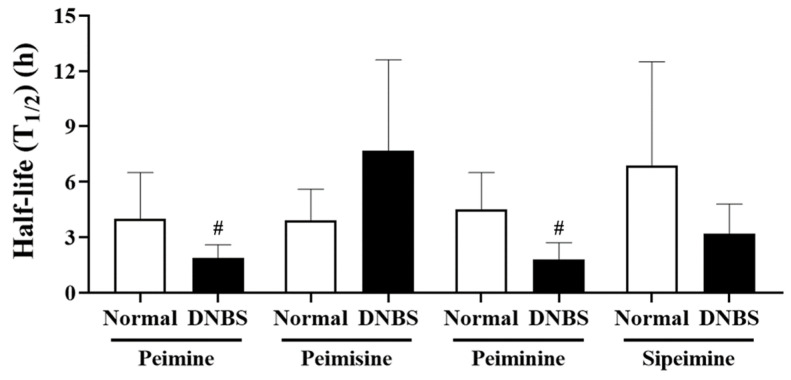
Half-life (T_1/2_) of peimine, peimisine, peiminine, and sipeimine after oral administration of *Fritillariae thunbergii* Bulbus (1 g/kg) to normal and 2,4-dinitrobenzene sulfonic acid (DNBS)-treated rats. Values: mean ± SD (*n* = 5). # *p* < 0.05, compared to normal rats treated with same dose by unpaired *t*-test.

Comparison of the elimination half-life of each substance (Figure 6), revealed that the other compounds, with the exception of peimisine, had a shorter (approximately two times shorter) half-life and mean residence time in the DNBS group. In particular, for peimine and peiminine, a shorter half-life was observed in the DNBS-treated group without significant differences in the extent of body exposure between the normal and DNBS groups.

## 3. Discussion

Pharmacokinetic study provides information about the effects of exposure to a compound administered within the body, and has recently furnished insights into the compounds found within the diverse, active constituents that contribute to the therapeutic effects of herbal medicines [15]. To achieve a deeper understanding of the possible clinical application of FTB on colitis management, four major steroidal alkaloids were selected as targets for FTB pharmacokinetic analysis, based on the ranking of their content in FTB. In this study, the pharmacokinetics of the four alkaloids were explored after oral FTB administration in a DNBS-colitis model to assess the impact of intestinal inflammation on the pharmacokinetics of steroidal alkaloids.

The anti-inflammatory efficacy of the four major steroidal alkaloids was explored using standard compounds, crucial for predicting the impact of pharmacokinetic changes on clinical outcomes under pathological conditions. Several studies have revealed the anti-inflammatory effects of the major steroidal alkaloids in FTB, among which peimine and peiminine have been reported to exhibit synergistic anti-inflammatory effects against LPS-induced acute lung injury [16,17]. In this study, as expected, all four steroidal alkaloids were found to suppress LPS-induced cytokine production in RAW264.7 cells, with peimisine showing the highest potency against IL-1β and TNF-α, and peiminine exhibiting the greatest potency against IL-6 among the alkaloids.

Following confirmation of the anti-inflammatory effects of the target alkaloids, the pharmacokinetics of the four alkaloids were explored after oral FTB administration in a DNBS-colitis model to assess the impact of intestinal inflammation on the pharmacokinetics of steroidal alkaloids. Although there are variations in the pathogenesis mechanism between DSS and DNBS-induced colitis, DNBS is widely used, along with DSS, to induce inflammation in the intestine because of its cost-effectiveness, rapid development of colitis, and localized damage to the distal colon [18]. DNBS is known to haptenize colonic and gut microbial proteins, making them immunogenic, and triggering both innate and adaptive immune responses in the host, resulting in significant inflammation and colon tissue damage. In the present study, taking into account serial blood collection points and cost-effectiveness in inducing colitis, we used a DNBS-induced rat model. Intrarectal DNBS successfully induced colitis in all treated rats, which was confirmed by the shortening and enlargement of the distal colon with significantly disrupted epithelial integrity.

The pharmacokinetic properties of the four steroidal alkaloids from FTB administered in this study were affected by DNBS-induced colitis conditions, with different patterns and to different extents. Among the steroidal alkaloids, peimisine was found to change greatly under colitis conditions, with an enhanced absorption rate and systemic exposure, which was confirmed by increased C_max_ and AUC values with statistical differences compared to normal conditions. In contrast, peimine, peiminine, and sipeimine showed no observable difference in systemic exposure in either the normal or colitis states. However, peimine, peiminine, and sipeimine exhibited shorter half-life under colitis than under normal conditions. The absorption of oral drugs is an intricate process that relies on the physiological conditions within the gastrointestinal tract as well as on the physicochemical properties of the drug, including its pharmaceutical formulation [14]. In general, intestinal transit times are shortened under colitis conditions and can alter the participation of intestinal microbiota in drug metabolism, thereby impacting drug bioavailability and elimination [19,20]. The exact reason for the shortened half-life without significant changes in systemic exposure of alkaloids under colitis conditions needs to be elucidated with further research, including investigations into the metabolic profiles of the target steroidal alkaloids.

The intestinal barrier, vital for separating the lumen from the external environment, can pose challenges for crossing larger molecules due to size restrictions and a greater probability of exclusion by the mucosal layer [21]. The tight junctions between intestinal epithelial cells are more easily passed by molecules below 500 Dalton, often resulting in better passive absorption [22]. The values related to the chemistry of the four major alkaloids, summarized in Supplementary Table S1, show that the molecular weights of the major alkaloids ranged from 427.6 to 471.7, which were smaller than 500 Dalton [23]. Another crucial factor for intestinal absorption is the drug’s lipophilicity, which must be sufficient for the drug to partition into a bilayer and remain un-charged. The four steroidal alkaloids reported a similar steroidal structure with a basic pKa. The pKa value of peimisine, which showed relatively significant changes under colitis conditions, was comparable to those of the other alkaloids, peiminine, and sipeimine, with a value of approximately 14 [23]. In contrast, while the partition coefficient (logP) values of the four alkaloids fell within the moderate range, peimisine exhibited a logP value of 2.7, whereas peimine, epiminine, and sipeimine had logP values of 4.1, 3.9, and 4.4, respectively [23]. A log P difference of 1 corresponds to a 10-fold difference in the partition coefficient. It is presumed that due to the relatively lower logP value of peimisine, the absorption process of peimisine is more likely to be influenced by the condition of the intestinal barrier when compared to other alkaloids, which may explain the substantial difference in systemic exposure to peimisine between the intact intestinal barrier in the normal state and compromised tight epithelial junctions in the colitis state. The lack of an intravenously administered group in this study hindered the precise assessment of the alterations in the volume of distribution and clearance rates of each alkaloid under colitis conditions. Moreover, due to an insufficient sample size, some factors made it difficult to provide pharmacokinetic interpretations based on statistical significance. Nonetheless, this study yielded valuable findings regarding shifts in drug exposure and changes in the elimination half-life, attributable to colitis, of specific alkaloids from FTB.

## 4. Materials and Methods

### 4.1. FTB Analysis

The hydroethanolic extract of FTB was obtained and authenticated by the Korea Institute of Oriental Medicine (Daejeon, Republic of Korea). The voucher specimen was deposited at the Korea Institute of Oriental Medicine. The extraction process is described in Lee et al. [11]. The reference standard was purchased from ChemFaces (Wuhan, China).

FTB content analysis was performed using ultra performance liquid chromatography–electrospray ionization–mass spectrometry (UPLC–MS/MS; Agilent Technologies Inc., Santa Clara, CA, USA). The chromatography separation was carried out with Eclipse Plus C18 2.1 × 150 mm, 3.5 μm (Agilent-technologies Inc.), which was maintained at a temperature of 40 °C. The mobile phase used was 0.1% formic acid in distilled water (buffer A) and acetonitrile (buffer B) at a flow rate of 0.250 mL/min. The elution gradient was as follows: 0–1 min, 97% A; 1–2 min, 97–85% A; 2–13 min, 85–50% A; 13–20 min, 50–0% A; 20–23 min, 0% A; 23–23.5 min, 0–97% A; 23.5–27.5 min, 97% A.

### 4.2. In Vitro Analysis of Anti-Inflammatory Activity of Steroidal Alkaloids

The RAW 264.7, a mouse macrophage cell line (American Type Culture Collection, Manassas, VA, USA), was grown in DMEM medium supplemented with 10% fetal bovine serum and 1% penicillin–streptomycin at 37 °C with 5% CO_2_.

To determine cell viability against the four steroidal alkaloids, peimine, peimisine, peiminine, and sipeimine, RAW 264.7 cells were seeded at a density of 5 × 10^4^ cells/well in 96-well plates and incubated overnight. Cells were then incubated with various concentrations of the four compounds (0, 0.78, 1.56, 3.13, 6.25, 12.5, 25, 50, and 100 μg/mL) for 24 h, respectively. Subsequently, 10 μL of EZ-Cytox (DoGenBio Co., Seoul, Republic of Korea) was added and incubated for 1 h. Absorbance was measured at 450 nm using an enzyme-linked immunosorbent assay reader (Bio-Rad Laboratories, Hercules, CA, USA). Cell viability was calculated by comparing the values measured with those of untreated cells.

To evaluate the anti-inflammatory effects of the active ingredients in FTB, the cells were seeded in 12-well plates at 3 × 10^5^ cells/mL and treated with 25 μg/mL of the four steroidal alkaloids, peimine, peimisine, peiminine and sipeimine, respectively, a concentration determined to be non-toxic according to the cell viability assay. LPS (Sigma-Aldrich, St. Louis, MO, USA) from *Escherichia coli* O111:B4 was used as a control at a concentration of 10 μg/mL. Cells were pretreated with the compounds for 24 h, followed by treatment with LPS for 4 h. After cells were harvested, total RNA was extracted using a total RNA extraction kit (Samjung Bioscience, Daejeon, Republic of Korea), and the extracted RNA was reverse transcribed into cDNA. The synthesized cDNA was subjected to quantitative polymerase chain reaction to measure the mRNA expression levels of cytokines using the following forward and reverse primers: IL-1β, forward, 5′-TGGACCTTCCAGGATGAGGACA-3′; reverse, 5′-GTTCATCTCGGAGCCTGTAGTG-3′; IL-6, forward, 5′-TACCACTTCACAAGTCGGAGGC-3′; reverse, 5′-CTGCAAGTGCATCATCGTTGTTC-3′; TNF-α, forward, 5′-TCTCATCAGTTCTATGGCCC-3′; reverse, 5′-GGGAGTAGACAAGGTACAAC-3′; GAPDH, forward, 5′-TTCACCACCATGGAGAAGGC-3′; reverse, 5′-TGAAGTCGCAGGAGACAACC-3′.

### 4.3. Pharmacokinetics of FTB in Normal and Colitis-Induced Rats

#### 4.3.1. DNBS-Induced Colitis Rat Model

Ten-week-old male Sprague–Dawley rats were obtained from Samtaco (Osan, Republic of Korea), quarantined, and acclimatized for one week before the experiments. All animal experiments were approved by the Animal Care and Use Committee of Chungnam National University (202304A-CNU-067). The rats were fasted for 14–16 h the day before administration of DNBS (Sigma-Aldrich). After the rats were anesthetized with isoflurane, the colitis group received a single dose of 30 mg/250 μL in 50% ethanol through a medical grade polyurethane catheter, injected rectally into the descending colon 8 cm from the anal sphincter. DNBS was administered slowly over 1 min, and the rats were kept supine in the Trendelenburg position for 3 min after injection to prevent leakage [24]. The normal group received only 50% ethanol, following the same procedure as described above. The pharmacokinetic study was performed four days after DNBS administration, and both the normal and DNBS groups were orally administered a single dose of 1 g/kg FTB. Blood samples were obtained from the tail vein at 0, 0.5, 0.75, 1, 2, 4, 6, 8, 10, 12, 24, and 30 h, and the rats were sacrificed after the last blood collection. The plasma samples obtained were centrifuged at 16,000× *g* for 15 min and stored at −80 °C until analysis.

Body weight was measured daily after the DNBS injection, and additional phenotype assessment was followed to confirm colitis induction including cecum to rectum length measurement. For histologic evaluation, colon tissue was fixed in 10% neutral-buffered formalin, embedded, sectioned at 4 μm thickness, and attached to slides. Tissue slides were stained with H&E (TissuePro Technology, Gainesville, FL, USA) and PAS (Sigma-Aldrich) staining.

#### 4.3.2. Sample Preparation and UPLC-MS/MS Analysis

Plasma concentrations were measured using UPLC-MS/MS (Agilent Technologies Inc.) in the same manner as described above. 100 μL of plasma was vortexed with 20 μL ammonia water and 800 μL ethyl acetate for 15 min and centrifuged at 16,000× *g* for 15 min. After evaporating 800 μL of the supernatant under nitrogen at 40 °C, 50 μL of 50% methanol was added. The mixture was then vortexed and centrifuged, and 5 μL of the final extract was used for analysis.

For the plasma concentration analysis, the limit of detection and limit of quantification (LOQ) were defined based on the concentration of the analyte, which generated signal-to-noise (S/N) ratios of 3 and 10, respectively, following the “Guideline on Bioanalytical Method Validation” for the Ministry of Food and Drug Safety in Korea (MFDS 2023) [25]. Among the active ingredients of FTB, peimine, peimisine, peiminine, and sipeimine were detected in concentrations above the LOQ —the multiple reaction monitoring conditions of each of them are summarized in Supplementary Table S2. The calibration curves were linear over the concentration range of 0 to 2000 ng/mL, with a correlation coefficient of R^2^ > 0.99. Precision and accuracy were evaluated by calculating the intra- and inter-day recoveries of quality control samples at three concentrations: 10, 50, and 100 ng/mL. The intra- and inter-day recoveries were within the range of 84.6 to 115.6%, and the coefficient of variation was less than 10%. Detailed validation results for the analysis method of peimine, peimisine, peiminine, and sipeimine are summarized in Supplementary Table S3.

#### 4.3.3. Pharmacokinetic Data and Statistical Analysis

The pharmacokinetic parameters were calculated based on a non-compartmental model using Monolix 2023R1 (Lixoft SAS, Simulations Plus Inc., Lancaster, CA, USA). The dosage of each component was calculated based on the content of the component within the FTB, and applied in the pharmacokinetics analysis. From the data analyzed, the C_max_ of FTB and the time to C_max_ (T_max_) were determined. Elimination half-life was obtained by linear regression of log-tranformed concentration data in the late phase. The AUC was estimated using the linear log-trapezoidal and linear up-log-down rules for the time of final concentration in each group.

All data are expressed as the mean ± standard deviation (SD). For statistical analysis, the Shapiro–Wilk test was used to confirm the normal distribution of the data, followed by an unpaired *t*-test for normally distributed and equal variances when comparing two groups. For comparisons of three or more groups, one-way analysis of variance (ANOVA) with Dunnett’s multiple comparisons test was used for normally distributed data. Detailed information is provided in the figure legends. Statistical significance defined as *p* value was less than 0.05 and 0.01, and GraphPad Prism version 9 (GraphPad Inc., La Jolla, CA, USA) was used.

## 5. Conclusions

In this study, we explored the possible impact of a colitis condition on the pharmacokinetics of active alkaloid components in FTB. Given the comparable anti-inflammatory effects exhibited by the four major steroidal alkaloids in FTB, it is suggested that the present alterations in substance pharmacokinetics due to colitis are unlikely to impact the overall anti-inflammatory effectiveness of FTB. However, for herbal medicines containing active compounds with different or even opposing efficacies, which may give rise to adverse effects, careful consideration of pharmacokinetic changes due to pathologic conditions is required. Notably, in the case of peimisine, our results suggest that the degree of drug exposure may be increased in the colitis condition. Further studies with the pure compound are needed to confirm the effectiveness and safety of the clinical application of peimisine.

## Figures and Tables

**Table 1:** Pharmacokinetic profiles (mean ± SD, *n* = 5) of peimine, peimisine, peiminine, and sipeimine after oral administration of *Fritillariae thunbergii* Bulbus (1 g/kg) to normal and 2,4-dinitrobenzene sulfonic acid (DNBS)-treated rats.

Parameter	Peimine		Peimisine	
	Normal	DNBS	Normal	DNBS
T_1/2_ (h)	4.0 ± 2.5	1.9 ± 0.7 #	3.9 ± 1.7	7.7 ± 4.9
T_max_ (h)	0.7 ± 0.2	1.0 ± 0.7	0.9 ± 0.2	1.5 ± 0.8
C_max_ (ng/mL)	356.2 ± 253.1	351.2 ± 149.5	655.5 ± 671.9	2330.5 ± 363.2 #
AUC_last_ (h∗ng/mL)	1311.8 ± 762.6	1076.9 ± 444.1	3959.3 ± 4738.6	16,895.4 ± 5782.1 #
AUC_%Extrap_obs (%)	6.7 ± 5.5	6.6 ± 6.6	5.3 ± 4.0	8.9 ± 11.6
AUMC_last_ (h∗h∗ng/mL)	4511.4 ± 2253.8	2819.7 ± 1408.9	23,522.8 ± 33,482.7	126,755.7 ± 54,141.2 #
MRT_last_ (h)	3.7 ± 0.8	2.7 ± 0.6 #	5.0 ± 2.0	7.3 ± 1.3 #
Parameter	Peiminine		Sipeimine	
	Normal	DNBS	Normal	DNBS
T_1/2_ (h)	4.5 ± 2.0	1.8 ± 0.9 #	6.9 ± 5.6	3.2 ± 1.6
T_max_ (h)	1.0 ± 0.2	1.3 ± 0.8	1.3 ± 0.5	2.4 ± 1.2
C_max_ (ng/mL)	227.9 ± 109.5	209.0 ± 51.1	64.7 ± 13.4	162.3 ± 97.0
AUC_last_ (h∗ng/mL)	1172.4 ± 392.3	888.8 ± 286.0	463.5 ± 232.8	764.0 ± 285.5
AUC_%Extrap_obs (%)	14.5 ± 15.7	3.3 ± 2.9	10.4 ± 8.6	11.0 ± 8.3
AUMC_last_ (h∗h∗ng/mL)	5302.0 ± 1844.3	3149.7 ± 1242.0 #	2675.8 ± 2026.3	3588.3 ± 1486.0
MRT_last_ (h)	4.7 ± 1.1	3.6 ± 0.6 #	5.4 ± 1.7	4.7 ± 0.6

# *p* < 0.05, compared to normal rats treated with same dose by unpaired *t*-test. AUC_last_, area under the curve from the time of dosing to the last measurable positive concentration; AUMC_last_, area under the first moment curve from zero to the last quantified sampling point time; AUC_%Extrap_obs, percentage of AUC_INF__obs due to extrapolation from T_last_ to infinity; C_max_, maximum plasma concentration; MRT_last_, mean residence time from the time of dosing to the time of the last measurable concentration; T_1/2_, half-life; T_max_, time to peak concentration.

## Data Availability

Data is contained within the article.

## Supplementary Materials

The following supporting information can be downloaded at: https://www.mdpi.com/article/10.3390/ph17081001/s1, Table S1: Values related to the chemical properties of peimine, peimisine, peiminine, and sipeimine. Table S2: Multiple reaction monitoring parameters for peimine, peimisine, peiminine, sipeimine, solanidine, hupehenine, and cyclopamine. Table S3: Validation results for the analysis method of peimine, peimisine, peiminine, and sipeimine.
