# Supplementary material for: Investigating Changes in Pharmacokinetics of Steroidal Alkaloids from a Hydroethanolic Fritillariae thunbergii Bulbus Extract in 2,4-Dinitrobenzene Sulfonic Acid-Induced Colitis Rats
Source: Pharmaceuticals (Basel). 2024 Jul 29;17(8):1001. doi: 10.3390/ph17081001 (PMC11357484; doi:10.3390/ph17081001)
Supplement: Supplementary file 1 [file pharmaceuticals-17-01001-s001.zip › pharmaceuticals-3116134-supplementary.pdf]

Supplementary Table S1. Values related to the chemical properties of peimine, peimisine, peiminine, and sipeimine.

	Peimine	Peimisine	Peiminine	Sipeimine
Molecular weights (Dalton)	431.7	427.6	429.6	471.7
Polar surface area (Å^2)	63.9	58.6	60.8	66.8
pKa	9.5	14.89	14.56	14.56
Partition coefficient (logP)	4.1	2.7	3.9	4.4

National Center for Biotechnology Information. PubChem Compound Summary. Available online: https://pubchem.ncbi.nlm.nih.gov.

Supplementary Table S2. Multiple reaction monitoring parameters for peimine, peimisine, peiminine, sipeimine, solanidine, hupehenine, and cyclopamine.

Compound	Precursor (m/z)	Production (m/z)	Fragmentor	Collision Energy (eV)	RT (min)	LOD (ng/mL)	LOQ (ng/mL)	Polarity
Peimine	432	432, 414	155	10, 30	7.4	0.2	0.6	$[M+H]^+$
Peimisine	428	428	155	10	7.1	0.2	0.6	$[M+H]^+$
Peiminine	430	430, 412	155	10, 40	7.9	0.02	0.05	$[M+H]^+$
Sipeimine	430	430	155	5	6.8	0.2	0.6	$[M+H]^+$
Solanidine	398	398	135	10	11.6	-	-	$[M+H]^+$
Hupehenine	416	416	135	10	8.6	-	-	$[M+H]^+$
Cyclopamine	412	412	135	10	9.9	-	-	$[M+H]^+$

LOD, limit of detection; LOQ, limit of quantification.

Supplementary Table S3. Validation results for the analysis method of peimine, peimisine, peiminine, and sipeimine.

Analyte	QC (ng/mL)	Day	Recovery			Inter-day		Intra-day	
			1	2	3	Mean recovery (%)	CV (%)	Mean recovery (%)	CV (%)
Peimine	10	1	84.6	94.5	101.5	93.5	8.5	97.5	5.7
		2	107.2	101.7	103.3	104.1	2.8		
		3	95.6	92.3	97.1	95.0	2.5		
	50	1	112.6	107.6	101.8	107.3	5.4	103.9	3.4
		2	100.4	113.3	98.0	103.9	8.2		
		3	101.4	100.8	99.2	100.5	1.1		
	100	1	97.0	98.2	99.5	98.2	1.3	100.4	3.1
		2	100.4	96.7	99.9	99.0	2.0		
		3	101.6	105.2	104.8	103.9	2.0		
Peimisine	10	1	99.1	101	100.2	100.1	1.0	94.3	6.8
		2	98.0	103.2	86.5	95.9	8.5		
		3	84.6	89.5	86.2	86.8	2.5		
	50	1	105.2	101.6	98.3	101.7	3.5	101.5	2.4
		2	100.3	99.3	111.9	103.8	7.0		
		3	97.5	101.3	98.4	99.1	2.0		
	100	1	98.7	99.6	100.4	99.6	0.9	100.6	2.2
		2	99.9	100.2	97.2	99.1	1.7		
		3	102.8	101.1	105.5	103.1	2.2		
Peiminine	10	1	99.4	91.7	93.8	95.0	4.0	92.2	2.4
		2	97.3	87.3	89.7	91.4	5.2		
		3	89.9	92.4	88.7	90.3	1.9		
	50	1	101.7	105.7	107.1	104.8	2.8	103.0	3.4
		2	102.5	106.5	106.4	105.1	2.3		
		3	99.4	101.8	96.2	99.1	2.8		
	100	1	99.6	98.7	98.3	98.9	0.7	100.5	2.8
		2	99.4	98.5	98.5	98.8	0.5		
		3	102.5	104.6	104.2	103.8	1.1		
Sipeimine	10	1	98.0	106.5	109.6	104.7	6.0	103.1	8.4
		2	107.1	115.6	109.4	110.7	4.4		
		3	97.4	92.9	91.8	94.0	3.0		
	50	1	103	101.9	99.3	101.4	1.9	99.3	4.7
		2	100.2	93.7	87.9	93.9	6.2		
		3	101.4	106.9	99.6	102.6	3.8		
	100	1	99.3	99.5	100.1	99.6	0.4	99.8	1.6
		2	99.9	101.4	102.9	101.4	1.5		
		3	102.8	94.9	97.1	98.3	4.1		

QC, quality control; CV, coefficient of variation.
